# Relationship between serum vitamin C and serum uric acid in people with different BMIs: results from the NHANES 2017–2018 and Mendelian randomization study

**DOI:** 10.3389/fnut.2024.1429123

**Published:** 2024-08-23

**Authors:** Jiajie Zhang, Hejun Jiang, Guanghui Fu, Zou Wu, Yukai Yao, Jie Sun

**Affiliations:** ^1^Department of Urology, National Children's Medical Center & Shanghai Children's Medical Center Affiliated to Shanghai Jiao Tong University School of Medicine, Shanghai, China; ^2^Shanghai Jiao Tong University School of Medicine, Shanghai, China

**Keywords:** serum uric acid, BMI, serum vitamin C, NHANES, Mendelian randomization

## Abstract

**Objective:**

To examine the association of overweight/obesity and serum vitamin C (serum VC) with serum uric acid (SUA) and to assess causality using Mendelian randomization (MR).

**Methods:**

4,772 participants from the National Health and Nutrition Examination Survey (NHANES), 2017–2018 were included in this study. Multivariate linear regression, variance inflation factor and quantile regression were used to analyze the relationships between overweight/obesity and serum VC and SUA levels. Secondly, Mendelian randomization (MR) was utilized to mitigate bias and prevent reverse causality in the observational study. Genetic variants associated with obesity (*N* = 13,848), vitamin C levels (*N* = 64,979) and serum uric acid levels (*N* = 343,836) were sourced from the most extensive genome-wide association studies (GWAS). The primary analytical method employed was inverse variance weighted (IVW).

**Results:**

Based on the observational study, BMI was positively associated with SUA (β = 0.06, 95% CI: 0.05 to 0.07, *p* < 0.001) and serum VC was negatively associated with SUA (β = −0.14, 95% CI: −0.23 to −0.04, *p* = 0.005). In individuals with overweight/obesity (BMI > =25), the negative effects of serum VC on SUA enhanced with increasing serum VC. High serum VC level (Q4 level, above 1.19 mg/dL) reduced SUA (β = −0.30, 95% CI: −0.47 to −0.14, *p* < 0.001) in individuals with overweight/obesity compared to low serum VC level (Q1 level, below 0.54 mg/dL). IVW-MR analysis revealed a significant association between SUA levels and genetically elevated levels of VC (β = −0.03, 95% CI: −0.06 to −0.00, *p* = 0.029) and obesity (β = 0.06, 95% CI: 0.04 to 0.07, *p* < 0.001).

**Conclusion:**

Cross-sectional observational analysis revealed that BMI exhibited a positive correlation with SUA levels and that serum VC was negatively correlated with SUA levels; moreover, moderate serum VC can reduce SUA, especially in individuals with overweight/obesity. There was evidence indicating a causal effect of VC and obesity on SUA. It highlights the importance of VC in the management of SUA levels, particularly in overweight/obese individuals. The findings might be helpful for the management of high SUA levels.

## 1 Introduction

Uric acid (UA) is a mild organic acid formed as a byproduct of purine metabolism (1). Hyperuricemia (HUA) arises from a disruption in UA production and elimination. Elevated levels of serum uric acid (SUA) or HUA are strongly associated with various diseases, such as chronic kidney disease (2), hypertension (3), gout (4), and cardiovascular disease (5). Epidemiological data on HUA are incomplete, and its prevalence varies widely worldwide. Recent analysis has shown that the global prevalence of HUA is approximately 20% in adults (6) and 14% in adolescents (7). According to the latest survey of the US population, HUA had a prevalence of 20.7% (8). Among Chinese adults aged 18–59 years, prevalence rates are 15.1% in males and 5.8% in females (9). Studying the clinical aspects of HUA-related risk factors is crucial for early detection, prevention, and management of HUA, gout, and related conditions. Current research is exploring the correlation among obesity, dietary habits, and SUA levels.

Obesity and HUA, along with their associated health concerns, are becoming increasingly severe clinical and public health challenges. Substantial evidence indicates that SUA is independently and positively associated with the risk of obesity and metabolic syndrome (10, 11). Previous observational studies have shown a positive correlation between obesity, BMI and SUA levels (12–15). The connection between SUA and obesity can be elucidated through various mechanisms. Past studies have demonstrated that elevated SUA levels may contribute to obesity by hastening hepatic and peripheral lipogenesis (16, 17).

Existing studies have shown that in addition to urico-lowering drugs, dietary factors play an essential role in managing elevated SUA levels (18). Several studies have shown that vitamin C (VC) intake is a dietary factor that reduces SUA levels. A cross-sectional study conducted in Korea found an inverse relationship between dietary VC consumption and SUA levels (19). Similarly, another dietary review study revealed that individuals with HUA had notably lower VC intake compared to controls (20). Furthermore, a meta-analysis of randomized controlled trials demonstrated that VC supplementation had significant SUA-lowering effects (21). However, contrasting findings emerged from a randomized controlled trial involving patients with gout. No clinically significant reduction in SUA levels was observed after administering a moderate daily dosage of 500 mg of VC over an 8-week period (22).

As mentioned earlier, both obesity and VC can influence SUA levels. Additionally, there exists a mutual interaction between VC and obesity. Previous studies have found a negative correlation between serum VC levels and obesity index such as BMI and waist-to-hip ratio (23, 24). VC acts as an antioxidant that neutralizes free radicals, thereby reducing oxidative stress and preventing obesity and its complications (25).

Previous research on the impact of VC and obesity on SUA levels has shown inconsistent results, likely due to variations in different study regions and samples. Moreover, so far, no studies have explored the combined effect of VC and obesity on SUA levels and their interactions in a large sample cohort.

To investigate the effects of serum VC and BMI on SUA levels, we first conducted an observational study utilizing data from the National Health and Nutrition Examination Survey (NHANES), a vital foundation for nutritional monitoring in the U.S. population (26). Mendelian randomization (MR) is a burgeoning epidemiological method. It employs genetic variables as proxies for exposure (such as VC and obesity) to gauge their causal effects on a specific outcome (such as SUA levels).

Therefore, the current study aimed to examine the impact of serum VC and BMI on SUA levels and to assess the causal relationships between serum VC and BMI and SUA levels. This was achieved based on observational data from the NHANES and genetic data accessible to the public within the framework of MR analysis.

## 2 Methods

### 2.1 Overall study design

The current study was carried out in two phases. In stage 1, using data deposited in the NHANES database, we conducted multivariable regression analysis to explore the correlation between serum VC and BMI with SUA. In the second phase, we evaluated the causal impact of genetically determined serum VC levels and BMI on SUA by MR analysis of summary statistical data from GWAS.

### 2.2 Study population in the NHANES

**Figure 1:**
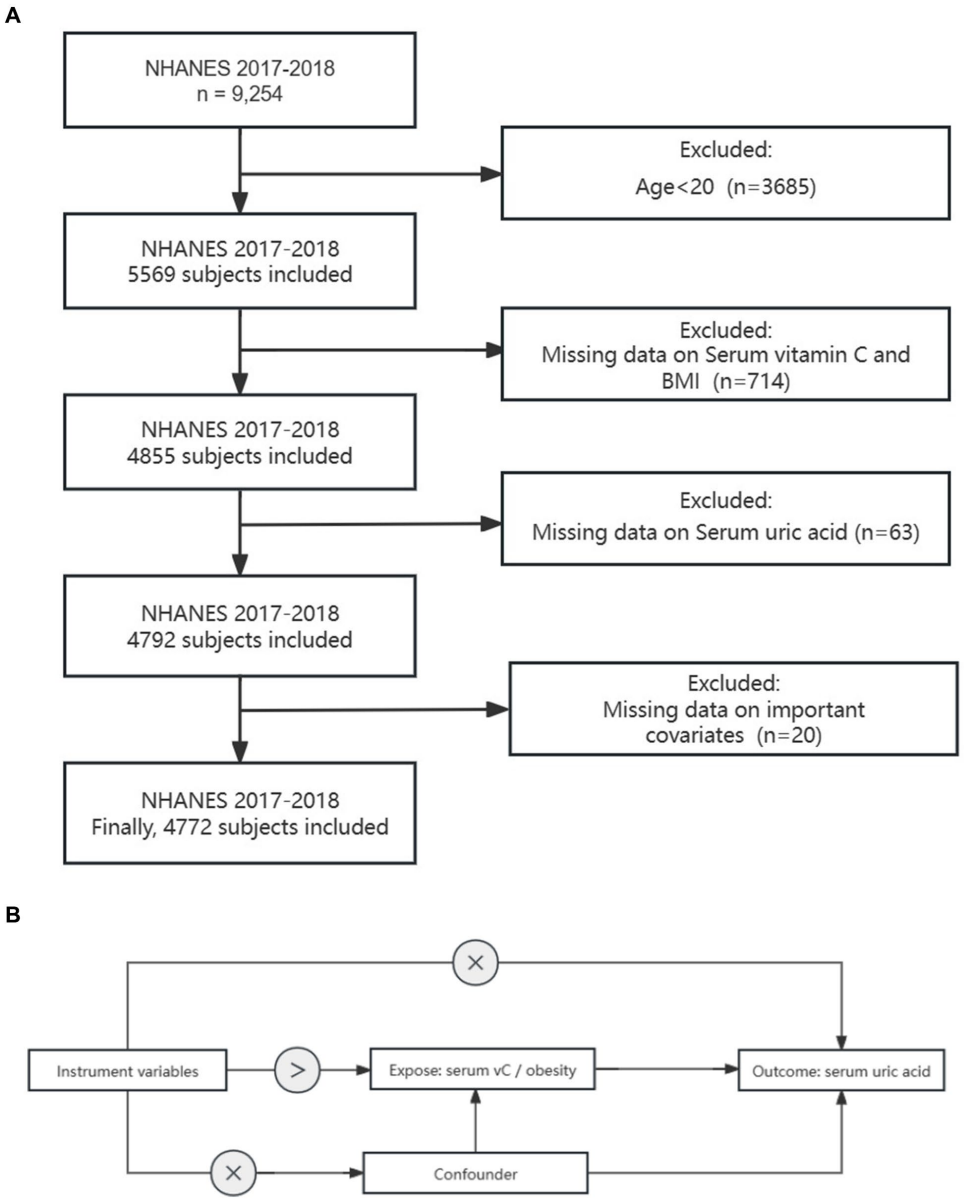
Study design overview: **(A)** Flowchart depicting the sample selection process from NHANES 2017–2018. **(B)** Explanation of the principles behind Mendelian randomization and the necessary assumptions to ensure unbiased estimation of causal effects.

The NHANES is a recurring biennial survey that is nationally representative in the US. The survey encompasses household interviews, physical examinations, and laboratory tests. The study protocol of the NHANES was approved by the National Centers for Health Statistics, and informed consent was obtained from all participants. We utilized publicly accessible NHANES survey data from the 2017–2018 cycle due to its comprehensive coverage of the exposure and outcome data required. A total of 4,772 participants were included in the analysis. Among all the participants, we excluded the following individuals: (1) individuals under the age of 20 (*n* = 3,685), (2) missing data on Serum VC and BMI (*n* = 714), (3) missing data on SUA (*n* = 63), (4) missing data on important covariates (*n* = 20). Consequently, 4,772 participants were ultimately included in the study (Figure 1).

### 2.3 Exposure and outcome variables in NHANES

Serum vitamin C levels (mg/dL) were measured using isocratic ultra-high performance liquid chromatography (UPLC). The peak area of the unknown quantity in serum samples was compared with that of the known quantity in the calibration solution to ascertain the quantitative data of vitamin C. Detailed descriptions of the laboratory measurement procedures and quality control protocols have been provided previously.^1^

BMI was computed using the formula weight (kg)/height (m)^2^. We treated BMI as a continuous variable and categorized weight status into underweight/normal weight (BMI under 25 kg/m^2^) and overweight/obesity (BMI 25 kg/m^2^ and above) (27).

Serum uric acid was the primary outcome variable of interest. The Roche Cobas 6000 (c501 module) method was employed to measure SUA levels during the period from 2017 to 2018.

The categorical variables of covariates considered in our analysis were as follows: gender (male or female), race/ethnicity (Mexican American, non-Hispanic White, non-Hispanic black, non-Hispanic Asian, other Hispanic, or other race), education levels (less than 9th grade, 9–11th grade, high school graduate/GED or equivalent, some college or AA degree, college graduate or above), poverty index, alcohol consumption (yes/no, based on at least 12 alcohol drinks in the last year), and smoking behavior (yes/no, based on at least 100 cigarettes in life), diabetes (with/without, based on HbA1c ≥ 6.5%), hypertension (with/without). The criteria for selecting covariates were determined based on prior published research and variables (28).

### 2.4 Genetic instruments for exposure and outcome variables in MR

For our MR analysis, we employed a two-sample design and utilized publicly accessible summary statistics derived from extensive GWAS datasets. For obesity, we obtained genetic data from a meta-analysis of encompassing 263,407 participants of European ancestry (29). As for vitamin C, we obtained genetic data from large-scale GWAS deposited in the UK Biobank (which encompassed 64,979 European individuals). SNPs used for SUA levels came from meta-analysis of UK Biobank cohorts and FinnGen (which included 628,000 European participants) (30).

### 2.5 Statistical analysis

We categorized serum VC into quartiles with quartile 1 as reference. Continuous variables were described using means ± standard deviation (SD), while categorical variables were presented as counts and weighted percentages. We compared baseline characteristics using chi-square, ANOVA, or the Wilcoxon rank-sum test depending on the data’s nature. To evaluate the relationship between serum VC, BMI, and SUA, we conducted univariate and multivariate linear regression models. Model 1 was the unadjusted model, Model 2 was adjusted for age, and Model 3 was the fully adjusted model (adjusted for age, gender, race, PIR, education attainment, smoking behavior, alcohol consumption, hypertension, diabetes) to reduce the impact of confounding factors and make the model more aligned with reality. The variance inflation factor (VIF) was utilized to assess multicollinearity among the variables. We also employed quantile regression to investigate the impact of serum VC on the correlation between BMI and SUA. Furthermore, we used linear regression to investigate whether the difference in SUA between different BMI groups disappeared under different serum VC levels.

As for two-sample MR analysis, the primary analytical method employed was inverse variance weighted (IVW). The genome-wide significance threshold was set at *p* < 1 × 10^−6^, the linkage disequilibrium (r2) threshold was set at 0.01, and a genetic distance of 10,000 KB was used to screen for instrumental variables without linkage effects. F statistics (F = β2/se2) were calculated to assess the strength of each instrument and ensure that each SNP is strongly associated with exposure. Additionally, we conducted the Cochrane Q test and utilized the MR-Egger intercept to examine possible heterogeneity and directional pleiotropy separately (31). The pleiotropy assessment was conducted to confirm the robustness of the MR analysis findings. A significance level of *p* < 0.05 indicated potential horizontal pleiotropy.

All statistical analyses were conducted using R software (version 4.3.2), and a significance level of *p* < 0.05 was deemed statistically significant.

## 3 Results

### 3.1 Baseline characteristic

Table 1 presents the baseline characteristics of the entire population and participants categorized by BMI. Our study included 4,772 subjects, with 2,482 (52%) females and 2,290 (48%) males. Individuals classified as overweight or obese exhibited a higher prevalence of diabetes. Notably, we observed that overweight and obese individuals tended to have elevated levels of SUA and lower levels of serum VC.

**Table 1:** Baseline characteristics stratified by BMI levels.

			BMI		
Characteristic	*N* ^a^	Overall, *N* = 4,772 (100%)^b^	Overweight/obese (25 or greater), *N* = 3,553 (74%)^b^	Underweight/normal (<25), *N* = 1,219 (26%)^b^	*p* Value^c^
Age (years)	4,772	48 (±17)	49 (±17)	45 (±18)	**<0.001**
Gender	4,772				**0.006**
Female		2,482 (52%)	1,816 (49%)	666 (58%)	
Male		2,290 (48%)	1,737 (51%)	553 (42%)	
Race	4,772				**<0.001**
Non-Hispanic White		1,677 (63%)	1,245 (62%)	432 (67%)	
Non-Hispanic Black		1,077 (11%)	830 (11%)	247 (9.6%)	
Non-Hispanic Asian		672 (5.6%)	366 (4.2%)	306 (9.8%)	
Mexican American		651 (8.9%)	558 (10%)	93 (5.0%)	
Other Hispanic		454 (7.0%)	373 (7.5%)	81 (5.4%)	
Other/multiracial		241 (4.7%)	181 (5.0%)	60 (3.6%)	
Education attainment	4,763				**0.019**
Less than 9th grade		399 (3.7%)	326 (4.0%)	73 (2.6%)	
9–11th grade		544 (7.5%)	390 (7.2%)	154 (8.5%)	
High school grade/GED		1,137 (27%)	840 (27%)	297 (27%)	
Some college or AA degree		1,547 (31%)	1,205 (32%)	342 (27%)	
College graduate or above		1,136 (31%)	784 (29%)	352 (35%)	
Smoking behavior	4,772				0.4
Non-smoker		2,762 (57%)	2,036 (57%)	726 (59%)	
Smoker		2,010 (43%)	1,517 (43%)	493 (41%)	
Alcohol consumption	4,042				0.6
Drinker		1,089 (22%)	806 (21%)	283 (23%)	
Non-drinker		2,953 (78%)	2,235 (79%)	718 (77%)	
Hypertension	4,249				0.2
High blood pressure		300 (5.7%)	243 (6.2%)	57 (4.1%)	
Non-high blood pressure		3,949 (94%)	2,920 (94%)	1,029 (96%)	
Diabetes	4,772				**<0.001**
Diabetes		699 (10%)	613 (17%)	86 (7%)	
Non-diabetes		4,073 (90%)	2,940 (83%)	1,133 (93%)	
Poverty index	4,155	3.10 (±1.64)	3.12 (±1.64)	3.02 (±1.66)	0.3
Serum VC (mg/dL)	4,772	0.90 (±0.49)	0.88 (±0.48)	0.97 (±0.53)	**0.001**
BMI (kg/cm^2^)	4,772	30 (±7)	33 (±6)	22 (±2)	**<0.001**
Serum uric acid (mg/dL)	4,772	5.38 (±1.44)	5.60 (±1.43)	4.74 (±1.27)	**<0.001**

a *N* not Missing (unweighted).

b Median (IQR) for continuous; *n* (%) for categorical.

c Chi-squared test with Rao & Scott’s second-order correction; Wilcoxon rank-sum test for complex survey samples.

Bold values indicate statistically significant p-values (*p* < 0.05).

### 3.2 The differences of SUA levels in the patterns of BMI-serum VC

**Figure 2:**
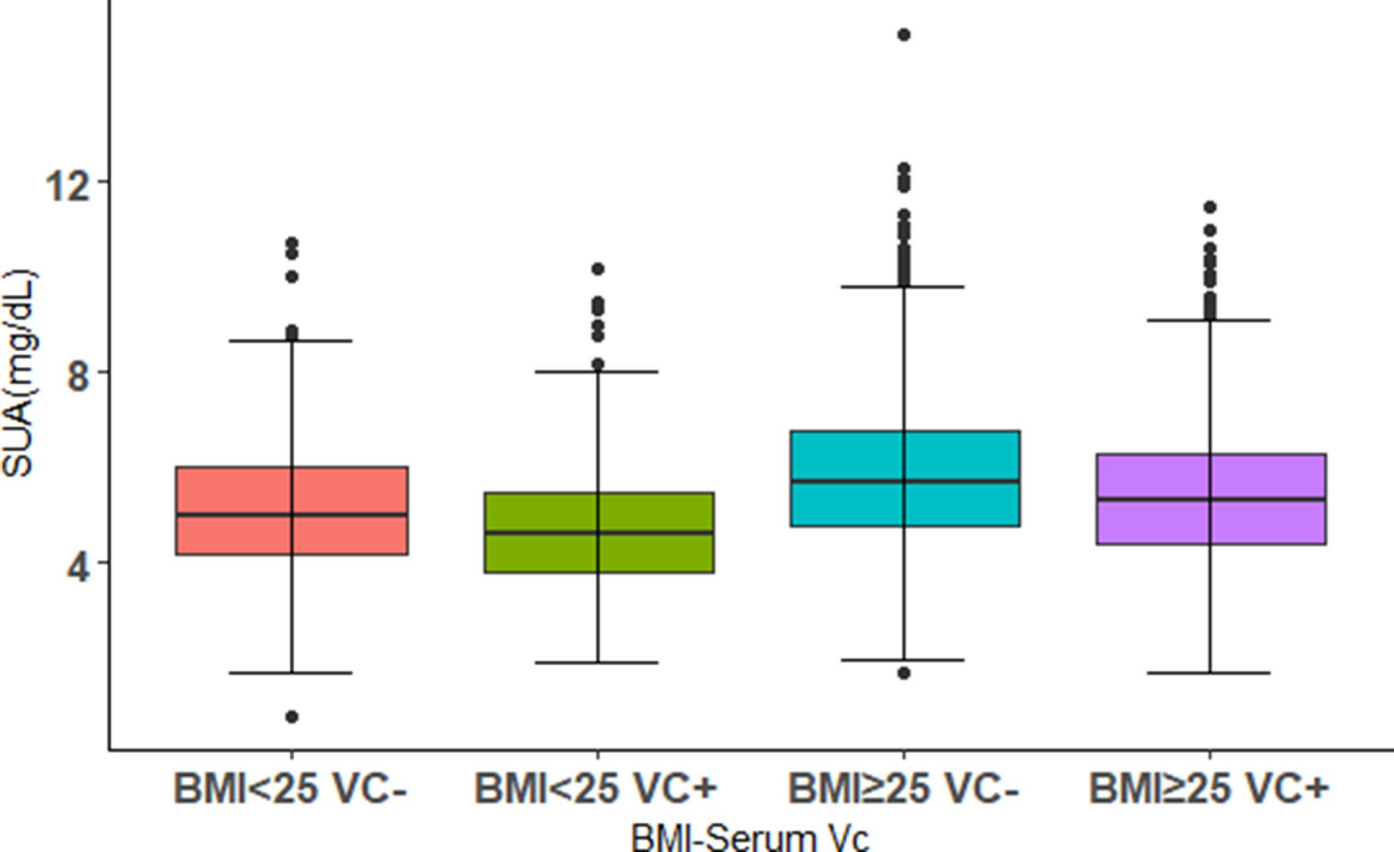
The distributions of SUA levels in different combinations of BMI levels and serum VC. Using 0.9 mg/dL as a threshold, VC+ and VC− were defined. The population was divided into four groups. Boxplot shows median (centerlines), lower/upper quartiles (box limits), and whiskers (the last data points 1.5 times the interquartile range (IQR) from the lower or upper quartiles).

The differences of SUA levels in the patterns of BMI-serum VC are shown in Figure 2. The BMI ≥ 25 VC− group had the highest SUA, whereas the lowest SUA were observed in the BMI < 25 VC+ group. Furthermore, we found that SUA in BMI < 25 VC+ group were lower than BMI < 25 VC− group. The same result was observed in the BMI ≥ 25 VC+ group and BMI ≥ 25 VC− group. All results are significant (*p* < 0.05).

### 3.3 Associations between serum VC, BMI levels and SUA

In the unadjusted model (Model 1), we observed a positive association between BMI and SUA (β = 0.05, 95% CI: 0.04 to 0.06, *p* < 0.001), whereas serum VC exhibited a negative association with SUA (β = −0.24, 95% CI: −0.34 to −0.13, *p* < 0.001). Upon adjusting for age (Model 2), we found that the positive BMI-SUA association and the negative serum VC-SUA association remained significant. In the fully adjusted model 3, the results were not considerably affected: BMI was positively associated with SUA (β = 0.06, 95% CI: 0.05 to 0.07, p < 0.001), while serum VC was negatively associated with SUA (β = −0.14, 95% CI: −0.23 to −0.04, *p* = 0.005). Multicollinearity was not present for all variables (Variance Inflation Factor, VIF < 5) (Table 2).

**Table 2:** Associations among BMI, serum VC and SUA using general linear regression.

	Model 1 (unadjusted)			Model 2 (age-adjusted)			Model 3 (fully adjusted)		
	Beta (95% CI)	*p* Value	VIF	Beta (95% CI)	*p* Value	VIF	Beta (95% CI)	*p* Value	VIF
Serum VC	−0.24 (−0.34,-0.13)	<0.001	1.002	−0.29 (−0.40, −0.18)	<0.001	1.115	−0.14 (−0.23, −0.04)	0.005	1.064
BMI	0.05 (0.04, 0.06)	<0.001	1.002	0.05 (0.04, 0.06)	<0.001	1.062	0.06 (0.05, 0.07)	<0.001	1.051

Model 2 was adjusted for age, while Model 3 adjusted for factors included in Model 2 along with additional adjustment for gender, race/ethnicity, education levels, poverty index, alcohol consumption, smoking behavior, BMI, serum VC, and hypertension.

### 3.4 Effects of different levels of serum VC on SUA in participants with different BMI levels

**Figure 3:**
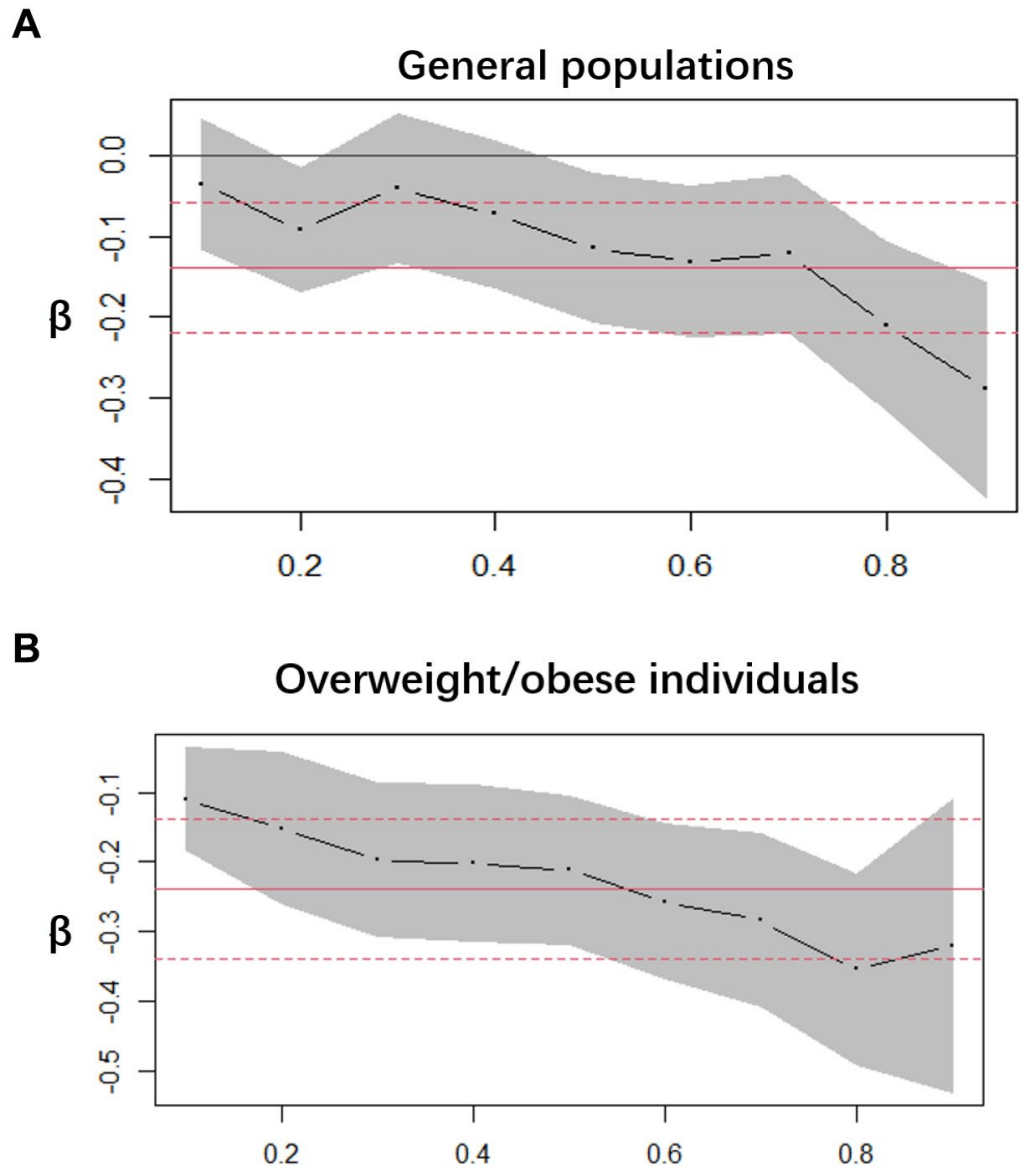
Decimal quantile regression of the association of serum VC and SUA. **(A)** General populations; **(B)** Overweight/obese individuals; the black line represents the smooth curve fit, while the gray transparent area indicates the 95% CI fit. The adjustments included gender, race/ethnicity, education levels, poverty index, alcohol consumption, smoking behavior, BMI, serum VC and hypertension.

We employed decimal quantile regression to evaluate the impact of various serum VC levels on SUA (Figure 3). In general populations, we found that the negative effects of serum VC on SUA (Figure 3A) were enhanced with the increase of serum VC and it was significant for all percentiles except 10th percentile and 30th percentile (The β of serum VC increased with the increasing serum VC). In comparison to general populations, the negative effects of serum VC on SUA were more significant in overweight/obese individuals (Figure 3B) and it was significant for all percentiles. The greatest negative effects were found in the 90th percentile in overweight/obese individuals.

**Figure 4:**
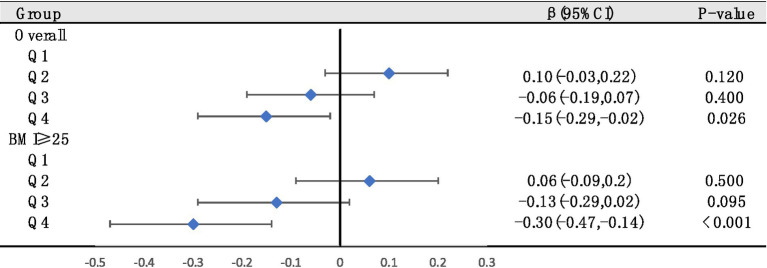
The relationships between serum VC and SUA were analyzed by dividing serum VC into quartiles in general populations (Q1, Q2, Q3, and Q4. Q1: serum VC ≤ 0.54 mg/dL; Q2: 0.54–0.89 mg/dL; Q3: 0.89–1.19 mg/dL; Q4: ≥1.19 mg/dL). Stratified analysis was conducted on overweight/obese individuals based on quartiles of the entire population as stratification nodes. Adjusted for gender, race/ethnicity, education levels, poverty index, alcohol consumption, smoking behavior, BMI and hypertension.

Next, to further investigate the effects of different serum VC levels on SUA, we classified serum VC by quartile and performed multiple linear regression in general populations (Figure 4). Using quartiles of the entire population as stratification nodes, stratified analysis was conducted on the overweight/obese individuals. Q1 was used as reference. The SUA of participants with Q4 level significantly decreased relative to Q1 intake in both general populations and overweight/obese individuals. The negative effect of Q4 serum VC on SUA (β = −0.30, 95% CI: −0.47 to −0.14, *p* < 0.001) was greater in overweight/obese individuals (β = −0.15, 95% CI: −0.29 to −0.02, *p* = 0.026). Other results were not significant.

**Figure 5:**
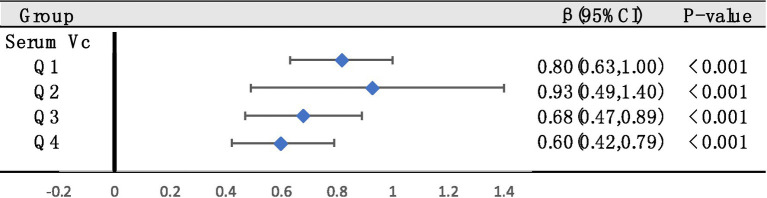
Stratified analysis to examine the relationships between overweight/obesity and SUA based on quartiles of serum VC across the entire population (These quartiles were denoted as Q1, Q2, Q3, and Q4. Q1: serum VC ≤ 0.54 mg/dL; Q2: 0.54–0.89 mg/dL; Q3: 0.89–1.19 mg/dL; Q4: ≥1.19 mg/dL). Adjusted for gender, race/ethnicity, education levels, poverty index, alcohol consumption, smoking behavior and hypertension.

Furthermore, we used multiple linear regression, exploring whether associations between overweight/obesity and SUA were still significant in different serum VC levels (Figure 5). We found that in all levels, the correlation between overweight/obesity and SUA still existed. However, the increase of serum VC levels had a tendency to abate the link between overweight/obesity and SUA.

### 3.5 Subgroup analysis

To verify whether the results of this study were robust, we conducted subgroup analysis based on demographic stratification (Table 3). Q4 level of serum VC decreased SUA in both males and females, and the inverse correlations were notably stronger in males than in females. The similar results were observed in the groups of 20–40 and > 60 years old. This effect was not significant in 40–60 years old group. No significant interaction was observed in all groups.

**Table 3:** Association between serum VC and SUA in overweight/obesity participants.

Group	β(Q4 to Q1)	95%CI	*p* Value
Gender			
Male (*N* = 1737)	−0.35	(−0.60,−0.10)	**0.007**
Female (*N* = 1816)	−0.27	(−0.49,−0.05)	**0.017**
Interaction			>0.05
Age			
20–40 (*N* = 962)	−0.49	(−0.80,−0.18)	**0.002**
40–60 (*N* = 1,185)	−0.23	(−0.53,0.07)	0.13
60 and above (*N* = 1,406)	−0.28	(−0.54,−0.01)	**0.039**
Interaction			>0.05

The *p*-value for interaction indicates the significance of differences among various gender and age groups. Covariates included gender, race/ethnicity, education levels, poverty index, alcohol consumption, smoking behavior and hypertension. Bold values indicate statistically significant p-values (*p* < 0.05).

### 3.6 MR of VC and obesity and SUA

**Figure 6:**
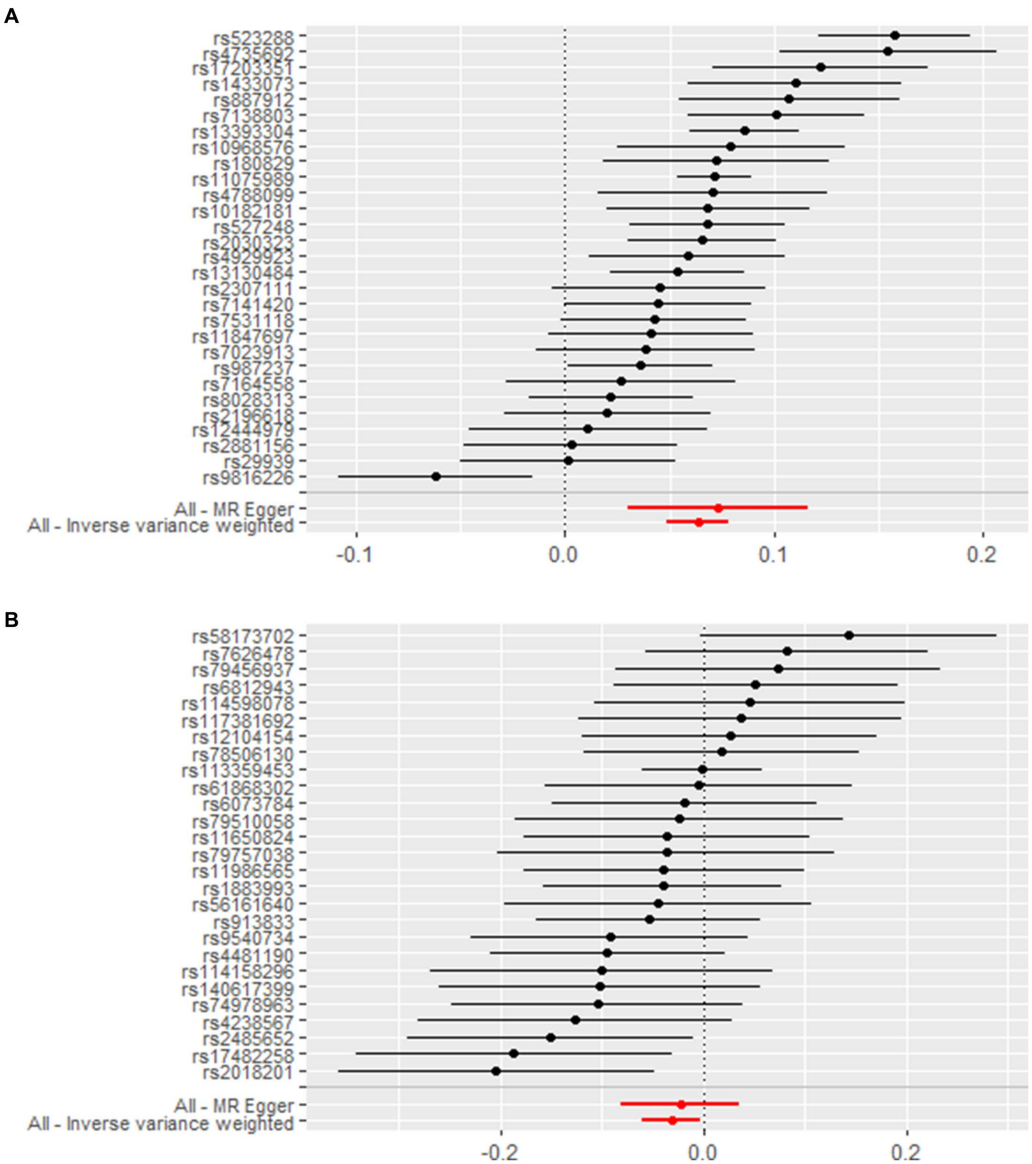
Forest plot demonstrates summary estimates of causal relationships between exposure and outcome. **(A)** Causal relationships between obesity and SUA; **(B)** causal relationships between VC and SUA.

We screened a total of 30 SNPs to serve as instrumental variables for obesity and 27 SNPs for VC. Each SNP had an *F*-value greater than 10. The IVW analysis revealed a causal link between obesity and SUA (OR = 1.07, 95% CI: 1.05 to 1.08, *p* < 0.001). Similar results were observed for the relationship between VC and SUA (OR = 0.97, 95% CI: 0.94 to 0.99, *p* = 0.029) (Figure 6). Additionally, we conducted a heterogeneity test to assess the stability of the IVW findings, which yielded a *p*-value of 0.156 for the IVW result concerning VC and SUA. We found proof of heterogeneity for the IVW result of obesity and SUA (*p* < 0.05) and it can be accepted as we utilized random-effects IVW approach for obesity and SUA. Pleiotropy analysis showed *p*-value of 0.639 and 0.747 (Table 4).

**Table 4:** Mendelian randomization was utilized to estimate the association between genetically predicted VC, obesity, and SUA.

Exposures	Outcome	Inverse-variance weighted		Pleiotrophy test		
		β (95% CI)	*p* Value	Intercept	SE (intercept)	*p*-Value
Obesity	SUA levels	0.06 (0.04,0.07)	<0.001	−0.014	0.008	0.14
Vitamin C	SUA levels	−0.03 (−0.06,−0.00)	0.029	−0.000	0.001	0.747

OR stands for odds ratio, and CI denotes confidence interval.

## 4 Discussion

Our study is the inaugural to employ the NHANES database for a cross-sectional analysis, merging it with the MR method to delve into the correlation among serum VC, BMI, and SUA. In our cross-sectional analysis, we found a negative association between serum VC and SUA, alongside a positive association between overweight/obesity and SUA. Furthermore, we demonstrated that the negative effects of serum VC on SUA enhanced with the increase of serum VC, this effect was more significant in overweight/obese individuals. Maintaining serum VC at Q4 (≥1.19 mg/dL) levels had the most beneficial impact on SUA control for both general populations and individuals with overweight/obesity. Our results remained robust in participants of different genders and age groups except 40–60 years old group. We further employed a two-sample MR analysis to investigate the causal relationship. MR analysis of the IVW method demonstrated a causal relationship between VC, BMI and SUA.

Dietary factors have always been an important research direction in the management of SUA levels and HUA. However, most studies mainly focus on the association between dietary intake of VC and SUA. Some studies have found that the total VC level in the body is determined by both diet and genes. Plasma VC level reaches a plateau at around 200 mg/day of VC intake, and the plasma VC level will not increase with higher doses of VC intake (32). Genetic variations in genes associated with redox homeostasis and VC transport contribute to individual differences. Therefore, analysis of the correlation between serum VC and SUA can better guide the diet management of individuals with elevated SUA levels and HUA patients. The molecular mechanisms underlying how VC reduces SUA level remain still unclear. Existing studies and viewpoints suggest that VC may affect UA excretion by competing with UA for absorption and transport in renal tubules (33) and by enhancing glomerular filtration rate to increase UA excretion (34). The specific molecular mechanism needs further investigation.

Furthermore, BMI has a significant impact on SUA levels. We found a positive correlation between BMI and SUA levels, consistent with previous epidemiological and clinical evidence. Metabolic syndrome represents a collection of risk factors for cardiovascular diseases, with obesity playing a crucially significant role in its manifestation (35). A meta-analysis suggested that patients with metabolic syndrome have elevated SUA levels, indicating SUA as a potential biomarker for early detection of metabolic syndrome (36). The relationship between obesity and SUA can be explained through various mechanisms: (1) Excess body fat in obesity may lead to increased SUA production and reduced excretion due to insulin resistance, impairing UA metabolism and potentially leading to HUA (37). (2) Adipokines such as adiponectin and leptin, secreted by adipocytes, may interact with SUA (38). (3) It has been reported in animal models that adipose tissue can generate and release UA via xanthine oxidoreductase and that this production was enhanced in obesity cases (39). It suggests that purine catabolism in adipose tissue could be enhanced in obesity, partly accounting for elevated SUA levels in obese individuals. In summary, obesity contributes to elevated SUA levels through increased UA production and reduced UA excretion. Besides, elevated SUA levels may contribute to obesity by speeding up hepatic and peripheral lipogenesis (16, 17). Dietary factors also have a mutual impact on obesity and SUA levels. High fructose content concurrently increases the risks of elevated SUA and obesity. Therefore, Mediterranean or DASH diets are more recommended compared to low purine diets, with an emphasis on increased VC intake (40). The significance of UA has been increasingly emphasized. UA is intricately linked with various organ systems, and obesity shares a bidirectional pathway with elevated SUA levels. Evidence suggests that obesity and elevated SUA may be mutually causative, thereby lowering SUA levels could be beneficial in preventing and treating obesity and its related metabolic syndrome (41, 42). Additionally, for managing HUA and gout, BMI control plays an important role. Understanding the intricate biological interplay between SUA and BMI is crucial in preventive medicine to evaluate their mutual influence.

In our investigation, we also identified a negative correlation between BMI and serum VC levels. Discrepancies have been noted in studies examining the link between BMI and serum VC (43–45), likely stemming from variations in study regions and sample demographics. A recent systematic review and meta-analysis revealed an inverse association between serum VC levels and BMI, with notable variations in mean serum VC levels observed between normal-weight individuals and those who are obese (46). Several mechanisms can explain the inverse association between BMI and serum VC levels. First, variations in dietary patterns among obese individuals may result in lower VC intake, consequently leading to reduced serum VC levels. Second, obesity is characterized by increased inflammation and oxidative stress, which accelerate the turnover of VC, thereby lowering its levels (47). Third, dysbiosis in the gut microbiota in obesity can inhibit VC absorption (48). VC, as an antioxidant, is believed to ameliorate diseases caused by inflammation and oxidative stress. An optimal serum VC level of 70 μmol/L is considered conducive to health, and VC supplementation may be employed to achieve this optimal circulating level (49). This is consistent with our study, we found maintaining serum VC at Q4 (≥1.19 mg/dL) levels had the best beneficial impact on controlling SUA levels for both general populations and individuals with overweight/obesity. According to a study endorsed by the European Food Safety Authority (EFSA), individuals with higher body weight are advised to consume increased quantities of VC to achieve adequate serum levels. The recommendation suggests adding 10 mg of VC per day for every 10 kg increase in body weight (50). These findings underscore the critical importance of maintaining sufficient serum VC levels in managing SUA levels among overweight and obese individuals. However, there is limited basic research on why VC reduces SUA levels especially in obese condition. It has been hypothesized that increased UA reabsorption in obese individuals may be an adaptive response of the kidneys to oxidative stress induced by fatty acids. VC is believed to reduce oxidative stress and thereby lower SUA levels, but experimental results in obese rat models have been inconclusive (51). Further research is needed to explore the significant SUA-lowering effect of VC in obese populations.

Subgroup analyses provided unique insights across different populations. Our results demonstrated robustness across genders and various age groups except 40–60 years old group. Nevertheless, the observed trend among individuals aged 40–60 aligns consistently with our overall study findings. This may be due to menopausal changes in women within this age group, where hormonal fluctuations may lead to increased SUA levels (52). The biological mechanisms underlying this phenomenon remain unclear, further longitudinal studies are needed for clarification.

Our study possesses several strengths. One notable advantage is the integration of an observational study with MR analysis, yielding consistent findings that enhance the reliability of our results. Our research underscores the significance of VC in regulating SUA levels, especially in overweight/obesity individuals. However, our study still has several limitations. Firstly, our study results are primarily based on populations from Europe and America, which limits the generalizability of our findings to other demographic groups. Secondly, despite efforts to adjust for potential confounding factors, not all of these factors could be accounted for. Thirdly, limitations in data detail arose from the survey’s lack of specific questions on gout diagnosis or medication use, including urate-lowering drugs and other medications influencing SUA levels. Future prospective cohort studies should encompass comprehensive data collection on factors influencing SUA levels to augment research depth. Despite these limitations, our findings could be valuable for managing and treating high SUA levels and gout, given the positive correlation between BMI and SUA levels, and the negative correlation of serum VC with SUA levels.

## 5 Conclusion

By combining a large, nationally representative observational study from NHANES with MR analysis, we discovered a positive correlation between BMI and SUA levels, while serum VC exhibited a negative correlation with SUA levels; maintaining serum VC at a sufficient level can reduce SUA, especially in individuals with overweight/obesity. There was evidence indicating a causal effect of VC and obesity on SUA levels. This discovery requires validation through additional well-designed prospective cohort studies. Moreover, it is essential to explore the underlying mechanisms further.

## Data Availability

The original contributions presented in the study are included in the article/supplementary material, further inquiries can be directed to the corresponding author.

## Footnotes

^1^http://cdc.gov/nchs/nhanes
